# VA-LOAM: Visual Assist LiDAR Odometry and Mapping for Accurate Autonomous Navigation

**DOI:** 10.3390/s24123831

**Published:** 2024-06-13

**Authors:** Tae-Ki Jung, Gyu-In Jee

**Affiliations:** Department of Electronic Engineering, Konkuk University, 120 Neungdong-ro, Gwangjin-gu, Seoul 05029, Republic of Korea; taeki222@konkuk.ac.kr

**Keywords:** Simultaneous Localization and Mapping (SLAM), sensor fusion

## Abstract

In this study, we enhanced odometry performance by integrating vision sensors with LiDAR sensors, which exhibit contrasting characteristics. Vision sensors provide extensive environmental information but are limited in precise distance measurement, whereas LiDAR offers high accuracy in distance metrics but lacks detailed environmental data. By utilizing data from vision sensors, this research compensates for the inadequate descriptors of LiDAR sensors, thereby improving LiDAR feature matching performance. Traditional fusion methods, which rely on extracting depth from image features, depend heavily on vision sensors and are vulnerable under challenging conditions such as rain, darkness, or light reflection. Utilizing vision sensors as primary sensors under such conditions can lead to significant mapping errors and, in the worst cases, system divergence. Conversely, our approach uses LiDAR as the primary sensor, mitigating the shortcomings of previous methods and enabling vision sensors to support LiDAR-based mapping. This maintains LiDAR Odometry performance even in environments where vision sensors are compromised, thus enhancing performance with the support of vision sensors. We adopted five prominent algorithms from the latest LiDAR SLAM open-source projects and conducted experiments on the KITTI odometry dataset. This research proposes a novel approach by integrating a vision support module into the top three LiDAR SLAM methods, thereby improving performance. By making the source code of VA-LOAM publicly available, this work enhances the accessibility of the technology, fostering reproducibility and transparency within the research community.

## 1. Introduction

Simultaneous Localization and Mapping (SLAM) is a process in which autonomous systems like robots or cars determine their location and simultaneously create a map of an unknown environment. This technology is a critical component in navigation systems such as unmanned aerial vehicles (UAVs) and autonomous driving vehicles. Visual Odometry (VO) and LiDAR (Light Detection and Ranging) Odometry are extensively used in these systems for tracking location and constructing maps. Understanding the unique strengths of each technology is vital for designing effective SLAM systems. 

Vision sensors, including monocular cameras, stereo cameras, and RGB-D cameras, collect high-frequency visual data (i.e., 30~60 Hz) and provide detailed spatial analysis. Monocular cameras alone face challenges in estimating depth, thus fusion with additional sensors is used to achieve more accurate Visual Odometry. For instance, studies [1,2,3,4] have enhanced the depth estimation accuracy of monocular cameras by integrating accelerometers and gyroscopes. Research [5,6] proposed new methods for performing Visual SLAM using stereo cameras. Stereo cameras can measure depth by aligning images from both lenses, allowing for more precise environmental mapping. To compensate for the limited depth range of stereo cameras, methods have expanded it using RGB-D cameras, as proposed in [7,8]. RGB-D cameras offer a more accurate and wider depth measurement range than stereo cameras but can face difficulties in outdoor environments due to their sensitivity to light.

LiDAR measures distances by detecting light reflected from objects, creating highly precise three-dimensional point clouds. Various studies [9,10,11,12,13,14] have proposed methods for Odometry and Mapping using LiDAR. However, as LiDAR operates at a low frequency (i.e., 10 Hz) and only provides 3D points and intensity data, spatial analysis can be challenging. While accurate mapping with LiDAR enables precise pose estimation, significant mapping errors may occur leading to serious pose estimation inaccuracies if sufficient descriptors are not provided.

Many studies propose methods that combine and complement the strengths of vision sensors and LiDAR sensors, which have contrasting characteristics. The approaches suggested in [15,16,17,18] involve extracting visual features from the vision sensor and measuring depth with the LiDAR sensor. Although these methods leverage the advantages of both sensors, the point cloud generated with the LiDAR sensor is less dense compared to the vision sensor, resulting in 3D–2D depth association errors. Particularly, these depth association errors become more pronounced with objects that are further away. Such errors can degrade the precision of LiDAR Odometry’s pose estimation. Moreover, vision sensors are highly dependent on environmental conditions such as weather, changes in lighting, shadows, and light reflections. Methods that use the vision sensor as the primary sensor in Visual–LiDAR Fusion are significantly affected by environmental changes, which can lead to substantial errors. In [19], deep learning is used to fuse the two sensors, while in [20], a method is employed to adjust the weights of each sensor’s measurements based on environmental conditions.

This paper proposes a new method that utilizes visual information from vision sensors to enhance the accuracy of LiDAR Odometry. We suggest a technique to reduce 3D–2D depth association errors and enable more precise pose estimation in LiDAR Odometry. By using only LiDAR features and assigning image descriptors to them, we enhance the uniqueness of the LiDAR points. Employing LiDAR as the primary sensor allows the system to maintain performance in LiDAR Odometry and Mapping even when vision sensors fail or environmental conditions change. This approach offers the advantage of maintaining the high precision of LiDAR sensors while minimizing the environmental limitations faced by vision sensors. To achieve this, we analyzed the performance of various open-source LiDAR Odometry methods using the KITTI dataset [21] and developed the Visual Assist LiDAR Odometry and Mapping (VA-LOAM) method, which integrates visual information into the top three methods with the lowest root mean square error (RMSE) on location.

To summarize, the main contributions of this work are fourfold:

(1) Visual Information Integration: This study proposes a new method that utilizes visual information collected from vision sensors to enhance the precision of LiDAR Odometry. This approach reduces 3D–2D depth association errors and enables accurate pose estimation in LiDAR Odometry. By integrating vision sensor data with LiDAR data, this method achieves better performance compared to traditional LiDAR Odometry. The rich environmental information provided via vision sensors complements the limitations of LiDAR, maintaining high accuracy even in complex environments;
(2) Enhanced LiDAR Odometry through Vision Sensor Support: We have clarified that this contribution focuses on using LiDAR as the primary sensor while utilizing vision sensors as a supplementary aid. Traditional methods that fuse LiDAR and vision sensors often rely on the vision sensor as the main sensor, which can fail in environments where vision sensors are weak (e.g., dark conditions and reflective surfaces). Our method ensures that in typical environments vision sensors assist in matching LiDAR feature points, improving accuracy. However, in challenging conditions for vision sensors, the system can operate using only LiDAR, maintaining the performance of traditional LiDAR-based Odometry. This approach ensures stable and consistent performance across various environments by leveraging the strengths of LiDAR while mitigating the weaknesses of vision sensors;
(3) Validation and Performance Improvement of VA-LOAM: This paper develops and validates the Visual Assist LiDAR Odometry and Mapping (VA-LOAM) method, which integrates visual information into existing LiDAR Odometry techniques. This method was tested using the publicly available KITTI dataset, demonstrating improved performance over existing LiDAR Odometry methods;
(4) Open-Source Contribution: By making the source code of VA-LOAM publicly available, this work ensures the reproducibility and transparency of the research across the community, enhancing the accessibility of the technology. This fosters collaboration and innovation in research and development.

## 2. Related Work

Vision sensors and LiDAR sensors are widely used for estimating the 6 degrees of freedom (6DOF) position and orientation. They are essential for accurately determining the position and orientation in UAV autopilot systems, robot SLAM, and autonomous vehicle navigation systems. There are three primary methods of SLAM that utilize these sensors: Visual SLAM, which uses only vision sensors; LiDAR SLAM, which uses only LiDAR sensors; and Visual–LiDAR SLAM, which integrates both vision and LiDAR sensors.

(1) Visual SLAM: Refs. [1,2,3,4,5,6,7,8] pertain to this method. LSD-SLAM [1] and SVO [2] match continuous images using photogrammetric consistency without preprocessing the sensor data. This approach is particularly useful in environments lacking distinct image features. Methods [3,4,5,6,7,8] extract local image feature points (edges, corner points, and lines) and track them to estimate their positional changes. By analyzing the camera’s motion and the feature points’ positional shifts, the distance to these points and the camera’s pose can be calculated. Additionally, image features are used to perform loop detection. ORB-SLAM [3] performs feature matching using fast features and brief descriptors. The pose is calculated based on matched feature points using the Perspective-n-Point (PnP) algorithm. VINS-Mono [4] is a tightly coupled sensor fusion method that uses visual sensors and IMUs. It performs visual–inertial odometry using tracked features from a monocular camera and pre-integrated IMU measurements. While accurate feature matching can be achieved through image descriptors, the process of estimating feature point depth involves significant errors. Research has been conducted using stereo cameras and RGB-D cameras to reduce these errors in depth estimation. TOMONO [5] and ENGEL [6] proposed methods using stereo cameras. TOMONO [5] introduced an edge point-based SLAM method using stereo cameras, which is particularly effective in non-textured environments where it detects edges and performs edge-based SLAM. ENGEL [6] improved SLAM accuracy by estimating pixel depth using a fixed-baseline stereo camera and motion from a multiview stereo. KERL [7] and SCHOPS [8] proposed RGB-D SLAM using entropy-based keyframe selection and loop closure detection;
(2) LiDAR SLAM: Refs. [9,10,11,12,13,14] apply to this method. This technique uses point clouds containing three-dimensional points and intensities, employing feature extraction and matching to estimate position. LiDAR provides accurate 3D points, enabling precise pose estimation. However, a lack of sufficient descriptors can lead to matching errors. Methods [10,11,12,13,14] have evolved from LOAM [9]. F-LOAM [14] offers faster processing speeds and lower memory usage, enabling real-time SLAM on lower-performance devices. A-LOAM [11] enhances accuracy by incorporating loop closure functionality and reducing mapping errors caused by obstacles. LeGO-LOAM [10] proposes a lightweight, terrain-optimized method for ground vehicles, classifying and processing the terrain accordingly. ISC-LOAM [13] addresses the issue of insufficient descriptors in LiDAR by proposing an intensity-based scan context, which improves performance in loop closure detection. LIO-SAM [12] tightly integrates LiDAR sensors with Inertial Measurement Units (IMUs), using pre-integrated IMU-derived estimated motion as initial values for point cloud corrections and LiDAR Odometry optimization;
(3) Visual-LiDAR SLAM: This technology is broadly classified into loosely coupled and tightly coupled approaches. The loosely coupled approach includes LiDAR-assisted Visual SLAM and Visual Assist LiDAR SLAM. LiDAR-assisted Visual SLAM [15,16,17,18] addresses one of the main issues in Visual SLAM—depth inaccuracies of image feature points—by correcting them with LiDAR data. While this greatly enhances the accuracy of feature points, the relatively low resolution of LiDAR data can lead to errors in 3D–2D mapping. To mitigate these issues, more precise data integration techniques are required. Visual Assist LiDAR SLAM utilizes LiDAR Odometry to estimate position and uses image data from the corresponding location to recognize the environment. It employs image feature points for loop closure detection, minimizing errors that may occur over time. The tightly coupled approach combines the advantages of LiDAR Odometry and Visual Odometry. In this method, rapid pose estimation from Visual-assisted Visual Odometry is refined through LiDAR Odometry. Although tightly coupled systems are robust, they require substantial computational resources;

Loosely Coupled Systems: These systems are relatively simple to implement and offer high accuracy. However, they can be vulnerable to sensor errors and changes in dynamic environments;

Tightly Coupled Systems: These systems are strong against uncertainty in sensor data and environmental changes, enabling precise position estimation. Nevertheless, they necessitate high-performance processing capabilities and sophisticated data integration techniques.

## 3. Preliminaries

### 3.1. Coordinate Systems

**Figure 1:**
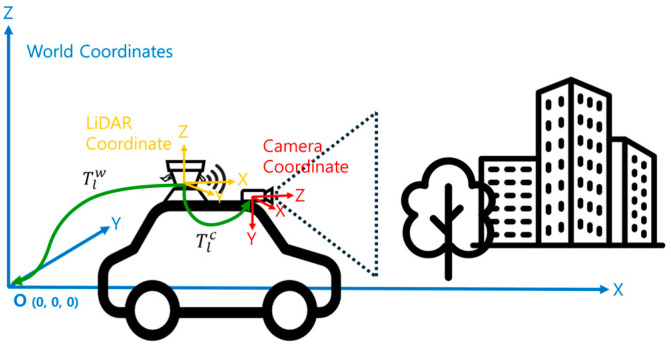
This illustration visually represents the process by which each sensor’s coordinate system is transformed into the world coordinate system. Data collected in each sensor coordinate system can be expressed in another sensor’s coordinate system or integrated into the world coordinate system through transformation matrices.

As seen in Figure 1, we utilize three coordinate systems: ${\left(\cdot \right)}^{w}$ denotes the world coordinate system, ${\left(\cdot \right)}^{c}$ denotes the camera coordinate system, and ${\left(\cdot \right)}^{l}$ denotes the LiDAR coordinate system. The world coordinate system provides a fixed reference frame and serves as the reference point for all other coordinate systems. The LiDAR and camera coordinate systems are defined based on the sensor’s position and orientation and can be transformed from the world coordinate system through rotations and translations. The origin of the world coordinate system coincides with the initial measurement position of the LiDAR coordinate system (odometry’s initial position). At the same time, $t_{i}$, the relationship between the camera coordinate system and the LiDAR coordinate system, can be expressed with the transformation matrix $T_{l_{i}}^{c_{i}}\in SE\left(3\right)$, which belongs to the special Euclidean group. $T_{l}^{c}$ represents the extrinsic parameters between the two sensors, consisting of a rotation matrix and a translation matrix. These extrinsic parameters, which indicate the relative position and direction between the two sensors, can be obtained through calibration [22].

(1) $$T_{l}^{c}=\left[\begin{array}{cc} R_{l}^{c} & t_{l}^{c}\\ 0 & 1 \end{array}\right]$$

(2) $$R\in SO\left(3\right),t\in R^{3},$$

### 3.2. Camera Projection Model

Data collected via LiDAR are measured as 3D points in the LiDAR coordinate system. Each coordinate, $X^{l}$, from the LiDAR point cloud is transformed into the camera coordinate system using a transformation matrix. Subsequently, these 3D coordinates are projected onto a 2D plane using the pinhole camera model matrix K. During this process, the depth information along the *Z*-axis is removed, resulting in 2D coordinates that correspond to the camera’s image plane. In Equation (5), *u* and *v* represent the coordinates in the image plane, *f* denotes the focal length of the camera, and *c* refers to the principal point of the camera.

(3) $$X^{l}={\left(x,y,z\right)}^{T}\in R^{3}$$

(4) $$X^{c}=T_{l}^{c}X^{l}$$

(5) $$x^{c}={\left(u,v\right)}^{T}=\left[\begin{array}{ccc} f & 0 & c_{x}\\ 0 & f & c_{y}\\ 0 & 0 & 1 \end{array}\right]T_{l}^{c}X^{l}\in R^{2}$$

## 4. Proposed Methodology 

### 4.1. System Overview

**Figure 2:**
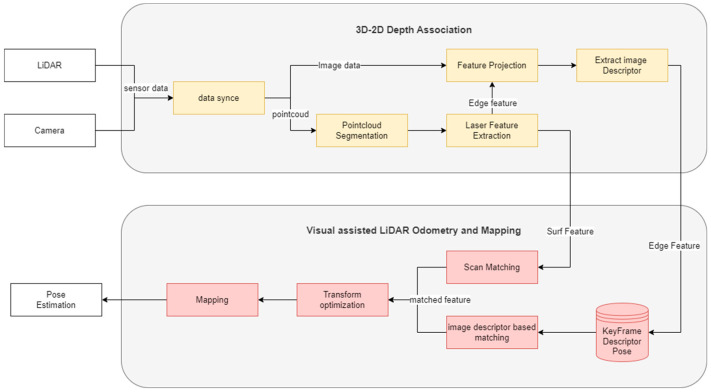
System flowchart for Visual Assist LiDAR Odometry and Mapping.

This study aims to enhance localization performance by integrating LiDAR Odometry and Mapping (LOAM) methods with a visual module, as shown in Figure 2. The overall system configuration is as follows. First, LiDAR data are used to detect LiDAR features (surf and edge). Next, the visual assist module uses images from the camera sensor to generate image descriptors for the LiDAR features. Then, to compute the motion displacement, the features detected in the previous frame are matched with those detected in the current frame. Edge features are matched using image descriptors, while surf features are matched using the conventional LiDAR Odometry method. If no matching data for the image descriptors are available, indicating a sensor failure or an environment where the camera sensor cannot operate, the edge features are also matched using the conventional LiDAR Odometry method, similar to the surf features. Since vision sensors are sensitive to environmental changes, LiDAR is utilized as the main sensor, with the vision sensor serving a supportive role. This configuration is implemented in a loosely coupled manner, allowing the visual module to enhance the performance of LiDAR Odometry under normal conditions, while maintaining its effectiveness even in environments where vision sensors are vulnerable.

### 4.2. Point Cloud Preprocessing

During the point cloud preprocessing stage, the detection of LiDAR features is a crucial task. The primary LiDAR features are categorized as edges and planars, based on the curvature calculations among adjacent point clouds. If the curvature exceeds a predefined threshold, the point is classified as an edge feature, indicative of significant local changes. Conversely, if the curvature does not surpass the threshold, it is classified as a planar feature, representing a relatively flat surface. This curvature-based extraction method plays a key role in environmental mapping and obstacle detection within LiDAR Odometry. This equation calculates the curvature c around a specific point $X_{\left(k,i\right)}^{l}$. The curvature is determined by considering the differences in distances between the point and its neighboring points. Higher curvature values indicate a more abrupt change in the geometric structure around the point.

(6) $$c=\frac{1}{\left|S\right||X_{\left(k,i\right)}^{l}|}||\sum_{j\in S,j\neq i}\left(X_{\left(k,i\right)}^{l}-X_{\left(k,j\right)}^{l}\right)||$$

### 4.3. Visual Assist LiDAR Feature

**Figure 3:**
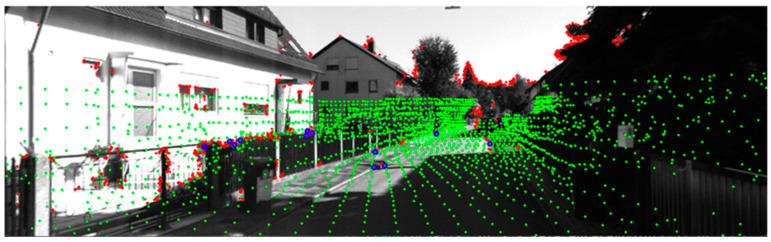
This figure demonstrates the depth association issues arising from the density differences between vision sensors and LiDAR sensors. The LiDAR point cloud is shown in green, image features in red, and corresponding features in blue.

Previous studies [15,16,17,18] have utilized LiDAR-assisted visual odometry based on image feature points. However, as illustrated in Figure 3, due to the density differences between vision sensors and LiDAR sensors, not all image feature points match with LiDAR 3D points. 

**Figure 4:**
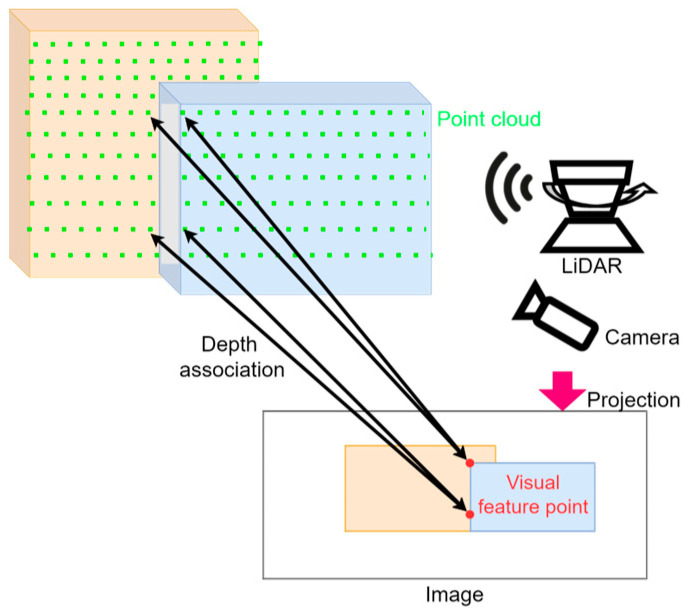
Illustration of depth association challenges in LiDAR-assisted Visual SLAM. This figure highlights the potential for multiple LiDAR point clouds to be projected onto a single image feature point.

As illustrated in Figure 4, depth association errors frequently occur at image features such as edges and corners. Image features are detected where there is a significant contrast from surrounding areas, particularly at edges or corners. LiDAR is not as dense as vision sensors, which makes it challenging to generate point clouds at precise locations like edges and corners. This discrepancy leads to incorrect matches between image feature points and LiDAR 3D points, resulting in errors in pose estimation. 

In this study, we propose a visual assist LiDAR feature matching method to resolve the depth association errors between image feature points and LiDAR 3D points. During the point cloud preprocessing phase, both edge and planar features are detected, and image descriptors corresponding to the edge features are extracted. These edge features, equipped with image descriptors, are defined as visual assist LiDAR features (VALFs). While planar features generally represent non-textured objects and do not provide significant differences in image feature vectors, edge features are suitable for image descriptor extraction due to their texture presence and curvature changes. Unlike LiDAR features, VALFs are detected based on three-dimensional terrain characteristics and can be matched through image descriptors.

**Figure 5:**
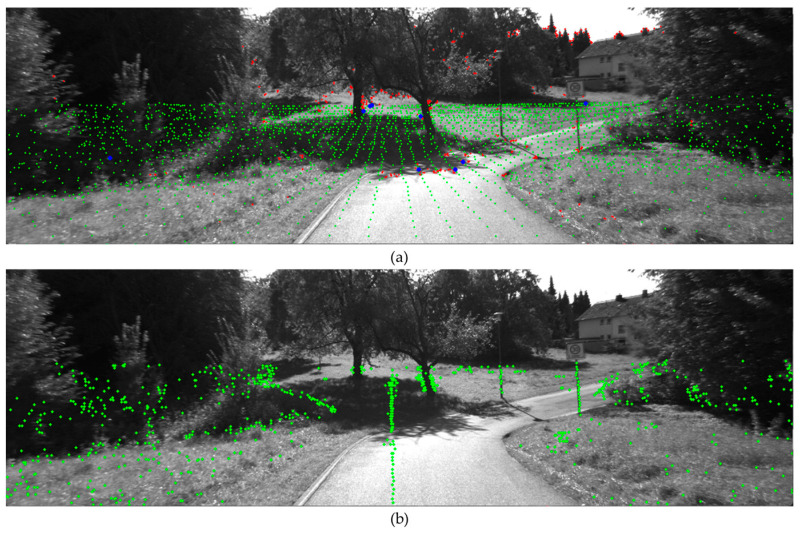
(**a**) Shows the 3D image features obtained by estimating the depth of image features using LiDAR point clouds, which is the conventional method. The LiDAR point cloud is shown in green, image features in red, and corresponding features in blue. (**b**) Illustrates the proposed VALF method presented in this paper.

Table 1 summarizes the advantages and disadvantages of various image descriptors. In this study, considering the presence of 200–300 LiDAR feature points projected onto urban area camera images, ORB and BRISK descriptors were selected for their balance of matching accuracy and processing time. Using Equation (6), edge features are extracted and then projected onto the camera image through Equation (5). If these features are present within the image region, visual descriptors are extracted to generate VALFs. Unlike traditional LiDAR edge/planar features, VALFs utilizes these descriptors to perform matching across consecutive frames, thereby enhancing the continuity and accuracy of the feature tracking process. We conducted experiments on 3D–2D depth association methods. Specifically, we tested the conventional method of estimating the depth of image features and the proposed method of applying image descriptors to LiDAR features. As seen in Figure 5, VALF generates a diverse and large number of 3D features compared to the conventional method of estimating the 3D depth of image features. The diversity and number of features are crucial factors in pose estimation.

**Figure 6:**
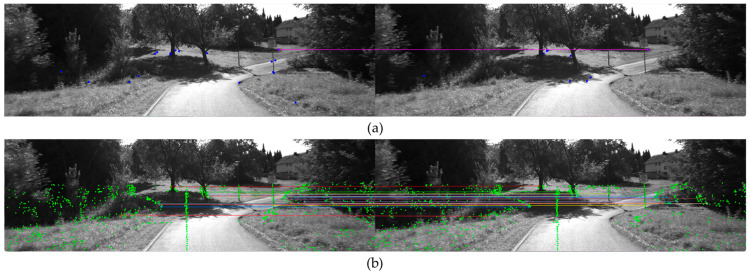
Shows the results of 3D feature matching between the current and previous frames. (**a**) Shows the matching of 3D image features, while (**b**) shows the matching of VALF features.

Figure 6 displays the results of matching the current frame and previous frame features obtained through the two 3D–2D depth association methods. As illustrated in Figure 6, it is evident that the number of matched features using VALFs is significantly higher.

### 4.4. Pose Estimation

Pose estimation utilizes VALFs ($F_{v}^{l}$), edge features $(F_{e}^{l}$), planar features ($F_{p}^{l}$), and a Global feature map ($M^{g}$). The Global feature map comprises an edge feature map ($M_{e}^{g}$), a planar feature map ($M_{p}^{g}$), and a VALF map ($M_{v}^{g}$). The edge and planar feature maps are stored in a 3D kd-tree structure, while the VALF map is stored as a vector containing descriptors and 3D points.

To estimate the optimal pose between the current frame and the Global map, the distance between matched Local and Global features is minimized. Local features consist of edge feature points $p_{e}^{l}\epsilon F_{e}^{g}$, planar feature points $p_{p}^{l}\epsilon F_{p}^{g}$, and VALF points $p_{v}^{l}\epsilon F_{v}^{g}$; Global features are composed of Global lines $p_{e}^{g}\epsilon M_{e}^{g}$, Global planars $p_{p}^{g}\epsilon M_{p}^{g}$, and Global VALFs $p_{v}^{g}\epsilon M_{v}^{g}$. The Gauss–Newton method, a nonlinear optimization technique, is employed to minimize the distances between these matched points in order to accurately estimate the optimal pose. This process involves point-to-line, point-to-planar, and point-to-point matching. The mathematical expressions for each matching type are as follows:

(7) $$\mathrm{Residual}\;\mathrm{for}\;\mathrm{edge}\;\mathrm{features}:\;f_{e}\left(p_{e}\right)=\frac{\left\|\left(Tp_{e}^{l}-p_{e_{u}}^{g})\times (Tp_{e}^{l}-p_{e_{v}}^{g}\right)\right\|}{\left\|p_{e_{u}}^{g}-p_{e_{v}}^{g}\right\|}$$

(8) $$\mathrm{Residual}\;\mathrm{for}\;\mathrm{planar}\;\mathrm{features}:\;f_{p}\left(p_{p}\right)=(Tp_{p}^{l}-p_{p}^{g})\cdot n_{p}^{g}$$

(9) $$\mathrm{Residual}\;\mathrm{for}\;\mathrm{VALFs}:\;f_{v}\left({Ρ}_{v}\right)=\left\|Tp_{v}^{l}-p_{v}^{g}\right\|$$

Global features are estimated by collecting points adjacent to the edge/planar feature points. Edge/planar features determine the position and direction of lines and planars by calculating their covariance matrices. The local smoothness of each feature point is used to compute weights that enhance accuracy by reflecting the importance of consistently extracted features during matching. VALFs, with their descriptors, facilitate finding matching local and global features. In environments where visual sensors are challenging to operate, optimization is performed using edge and planar features when VALFs do not match, and using VALFs and planar features when VALFs match. The optimization equations are as follows:

(10) $$\mathrm{When}\;\mathrm{VALFs}\;\mathrm{do}\;\mathrm{not}\;\mathrm{match}:\;\underset{T_{k}}{\min}\sum f_{e}\left(p_{e}\right)+\sum f_{e}\left(p_{p}\right),\;\mathrm{if}\;\mathrm{non}-\mathrm{matched}\;p_{v}$$

(11) $$\mathrm{When}\;\mathrm{VALFs}\;\mathrm{match}:\;\underset{T_{k}}{\min}\sum f_{v}\left(p_{v}\right)+\sum f_{e}\left(p_{p}\right),\;\mathrm{if}\;\mathrm{matched}\;p_{v}$$

Optimization does not simultaneously use all three types—VALFs and edge and planar features—due to the significant difference in the number of VALFs and edge features. LiDAR features cover a full 360-degree range, but cameras only cover the front, leading to a narrower field of view. Many edge features projected from the LiDAR onto the image fall outside the camera’s Field of View (FOV), resulting in a discrepancy in the number of points between VALFs and edge features. 

## 5. Experimental Setup and Validation

We employed the KITTI dataset [21] to evaluate the performance of Visual Assist Lidar Odometry and Mapping (VA-LOAM). The odometry benchmark from this dataset was used as the test set, initially experimenting with algorithms such as F-LOAM [14], A-LOAM [11], LeGO-LOAM [10], ISC-LOAM [13], and LIO-SAM [12]. From these, the top three performing algorithms were selected, and further experiments were conducted by integrating our proposed Visual Assist module. The accuracy of each experiment was quantitatively analyzed using the root mean square error (RMSE) as a measure of positional error.

### 5.1. Evaluation on Public Datasets

We conducted a performance evaluation of both traditional LiDAR-based Odometry and Mapping methods and the enhanced Visual Assist Lidar Odometry and Mapping (VA-LOAM) using the renowned KITTI dataset [21], which is notable for its road driving scenarios. This dataset comprises sensor data collected from vehicles equipped with stereo and mono cameras, Velodyne HDL-64 LiDAR, and high-precision GPS/INS, capturing LiDAR point clouds and camera images at a frequency of 10Hz. As shown in Table 2, the tests used the odometry benchmark from the KITTI training set, which contains ground truth position data. This evaluation aimed to compare the discrepancies between the positions estimated via each method and their ground truth positions. The dataset features a variety of environments, ranging from urban areas densely packed with buildings to rural areas lush with vegetation, and highways devoid of nearby objects. It also includes scenarios both with and without loop closures.

### 5.2. Performance of LiDAR Odometry and Mapping

We conducted a comparative analysis of existing LiDAR Odometry algorithms on the KITTI dataset [21], including F-LOAM [14], A-LOAM [11], LEGO-LOAM [10], ISC-LOAM [13], and LIO-SAM [12]. We compared their performances both with and without the integration of loop closure detection. In Table 3 and Table 4, the numbers highlighted in bold represent the algorithms that achieved the highest performance in each dataset sequence. Using only LiDAR for odometry, F-LOAM [14] and ISC-LOAM [13] showed equivalent performance. A-LOAM [11] did not exhibit the best performance in any of the datasets, while ISC-LOAM [13] performed well in 8 out of 13 datasets, LEGO-LOAM [10] in 2, and LIO-SAM [12] in 3. The RMSE of LiDAR Odometry was evaluated in the absence and presence of loop detection in the KITTI odometry dataset.

### 5.3. Performance of Visual Assist LiDAR Odometry and Mapping

In our performance comparison experiment using the KITTI dataset [21], we enhanced the top-performing methods—LEGO-LOAM [10], ISC-LOAM [13], and LIO-SAM [12]—by integrating a visual module, resulting in the development of VA-LEGO-LOAM, VA-ISC-LOAM, and VA-LIO-SAM. Each modified method maintains its original point cloud processing capabilities while incorporating an additional visual assist Lidar feature (VALF), which utilizes image descriptors to further refine the estimation of position and orientation. Table 5 and Table 6 detail the comparative performance of these enhanced methods against their original counterparts, showcasing the benefits of integrating the visual module.

**Figure 7:**
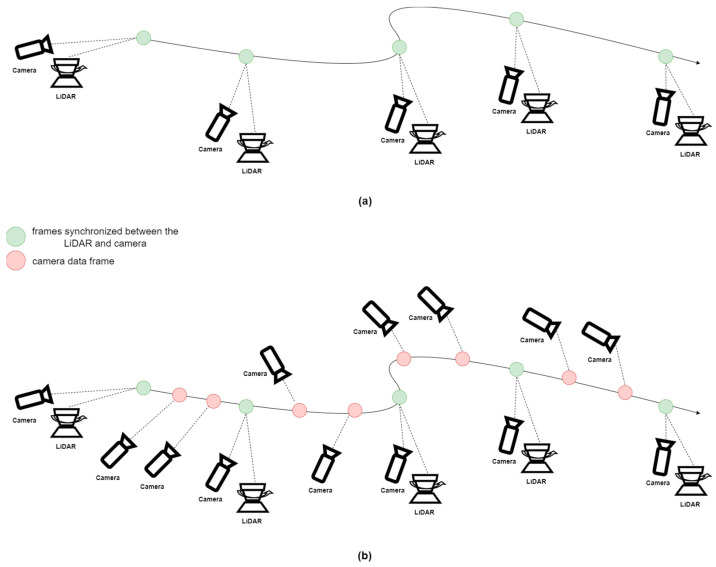
(**a**) shows the KITTI dataset consisting of synchronized sensor data, while (**b**) represents a typical asynchronous data system.

Table 7 and Table 8 show the improvement rates in position accuracy for each method, demonstrating a reduction in RMSE. These figures indicate that position estimation accuracy has improved and highlight the effectiveness of the visual module in reducing position errors under various conditions. Specifically, VA-LEGO-LOAM showed an average reduction of 7.02%, VA-ISC-LOAM showed 12.43%, and VA-LIO-SAM showed 3.67%. Examining each data sequence, in most cases, the visual assist module provided missing descriptors in the existing LiDAR feature matching process, thereby enhancing LiDAR feature matching performance and consequently improving position estimation accuracy. However, in some instances, performance degradation occurred. This was due to incorrect matching during the VALF process. In the KITTI dataset, as shown in Figure 7a, significant changes between the previous frame image and the current frame image occur due to the synchronization of the vision sensor and the LiDAR sensor, resulting in VALF matching errors. For example, if the vision sensor’s output frequency is 30Hz, like most vision sensors, and the LiDAR sensor’s output frequency is 10Hz, as illustrated in Figure 7b, there can be two additional images between LiDAR data frames. This allows for improved VALF matching through image-based VALF tracking. Future research will aim to address this issue by applying this method.

**Figure 8:**
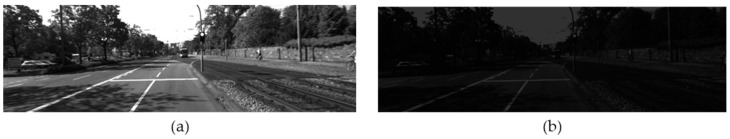
(**a**) is the daytime image, and (**b**) is the night-time image.

Table 9 presents the results of experiments conducted under varying environmental conditions. The test environments include daytime, night-time, and camera sensor failure. Night-time and camera sensor failures are challenging conditions for the visual assist module. The night-time data were generated by reducing the brightness of the original daytime image data and applying image processing, as illustrated in Figure 8. We tested VA-LEGO LOAM, VA-ISC LOAM, and VA-LIO-SAM, which integrate the sensor fault visual assist module. While the performance in night-time conditions was lower compared to daytime, the error did not increase significantly. This is attributed to the feature matching performance; although there is a difference in feature matching performance between images with a large time gap, the impact on feature matching between images with a small-time gap is minimal. Additionally, in cases where the camera sensor failed, the LiDAR Odometry performance remained stable. This is because the visual assist module cannot operate without image-based descriptors, but LiDAR-based Odometry can still be performed without image descriptors, maintaining the original LiDAR Odometry performance. This result demonstrates the advantages of a loosely coupled approach in sensor fusion, showing that using LiDAR as the main sensor, compared to the conventional method of obtaining depth using visual features, provides robustness against camera sensor failures and environmental changes.

## 6. Conclusions 

This paper provides several significant contributions. Firstly, it proposes a novel method to reduce 3D–2D depth association errors and enable accurate pose estimation in LiDAR Odometry by utilizing only LiDAR features instead of image key points and enhancing their uniqueness through image descriptors. Furthermore, it demonstrates the capability to maintain performance even in the event of vision sensor failure or environmental changes by leveraging LiDAR as the primary sensor. Through evaluations on the KITTI dataset [21], the top three methods with the lowest position RMSE are selected to develop Visual Assist LiDAR Odometry and Mapping (VA-LOAM), which evolves into subversions such as VA-LeGo-LOAM, VA-ISC-LOAM, and VA-LIO-SAM. The results of RMSE reduction in each version clearly indicate the potential of visual assistance modules to enhance LiDAR Odometry performance. This research lays a crucial foundation for the advancement of precise mapping and localization techniques using LiDAR and visual sensor data and provides broader research and application possibilities by making these methods publicly available.

## Figures and Tables

**Table 1:** Advantages and disadvantages of image descriptors.

Descriptor	Advantages	Disadvantages
SIFT	Scale invariance, rotation invariance, robustness to illumination changes	Slow computation speed
SURF	Performance similar to SIFT Faster speed	Vulnerable to specific lighting conditions
ORB	Fast speed–Real-time processing Low computational cost	Slightly lower accuracy
BRISK	Shares characteristics with ORB Superior performance in texture-rich images	Vulnerable to specific deformations
AKAZE	Performance similar to SIFT Fast speed	Vulnerable to specific textures
Deep Learning-based Descriptors	High accuracy	Requires large amounts of training data and computation

**Table 2:** This table presents the various environments and driving distances that comprise the KITTI dataset.

Dataset	Seq No.	Data Name	Environment	Path Len. (m)
KITTI Odometry Set	1	2011_09_30_drive_0016	Country	397
	2	2011_09_30_drive_0018	Urban	2223
	3	2011_09_30_drive_0020	Urban	1239
	4	2011_09_30_drive_0027	Urban	695
	5	2011_09_30_drive_0033	Urban + Country	1717
	6	2011_09_30_drive_0034	Urban + Country	919
	7	2011_10_03_drive_0027	Urban	3714
	8	2011_10_03_drive_0034	Urban + Country	5075
	9	2011_10_03_drive_0042	Highway	4268
KITTI Raw Data Set	1	2011_09_26_drive_0001	Urban	107
	2	2011_09_26_drive_0005	Urban	69
	3	2011_09_26_drive_0009	Urban	332
	4	2011_09_26_drive_0011	Urban	114

**Table 3:** The root mean square error (RMSE) of LiDAR Odometry was evaluated without loop detection in the KITTI odometry dataset.

Dataset	Seq No.	F-LOAM [14]	A-LOAM [11]	LEGO-LOAM [10]	ISC-LOAM [13]	LIO-SAM [12]
KITTI Odometry Set	1	**2.67**	5.00	3.65	**2.67**	5.78
	2	10.89	113.08	**9.53**	10.90	10.25
	3	9.25	21.28	**4.91**	9.25	21.34
	4	1.85	5.94	1.63	1.85	**1.55**
	5	**17.79**	124.16	22.94	**17.79**	20.08
	6	**8.67**	61.02	17.85	**8.67**	12.05
	7	**19.76**	182.32	171.39	**19.76**	fail
	8	**31.30**	183.91	294.73	**31.30**	41.76
	9	**145.49**	261.86	373.20	**145.49**	167.86
KITTI Raw Data Set	1	1.70	5.70	5.04	1.70	**1.22**
	2	1.53	1.77	1.76	1.53	**1.51**
	3	**5.86**	6.15	13.4	**5.86**	7.05
	4	**1.27**	3.66	2.2	**1.27**	1.71

Note: The numbers highlighted in bold indicate the algorithm that achieved the highest performance on each dataset sequence.

**Table 4:** The root mean square error (RMSE) of LiDAR Odometry was evaluated with loop detection in the KITTI odometry dataset.

Dataset	Seq No.	F-LOAM [14]	A-LOAM [11]	LEGO-LOAM [10]	ISC-LOAM [13]	LIO-SAM [12]
KITTI Odometry Set	1	**2.67**	2.70	3.74	**2.67**	5.77
	2	10.89	10.43	**7.61**	10.89	8.40
	3	9.25	8.46	**4.92**	9.25	19.02
	4	1.85	1.62	1.76	1.85	**1.50**
	5	**17.79**	18.71	22.93	**17.79**	20.15
	6	**8.67**	9.82	18.53	**8.67**	11.35
	7	**19.76**	20.77	158.65	**19.76**	fail
	8	**31.30**	101.64	275.88	**31.30**	39.79
	9	**145.49**	166.58	376.63	**145.49**	166.19
KITTI Raw Data Set	1	1.70	5.70	5.04	1.70	**1.22**
	2	1.53	1.77	1.76	1.53	**1.51**
	3	**5.86**	6.15	13.4	**5.86**	7.05
	4	**1.27**	3.66	2.2	**1.27**	1.71

Note: The numbers highlighted in bold indicate the algorithm that achieved the highest performance on each dataset sequence.

**Table 5:** Evaluation of RMSE for LiDAR Odometry with the proposed visual aid module (without loop detection). The visual aid module was incorporated into the three LiDAR odometers that exhibited the most favorable performance in the Kitti test set.

Dataset	Seq No.	LEGO-LOAM [10]	VA-LEGO-LOAM	ISC-LOAM [13]	VA-ISC-LOAM	LIO-SAM [12]	VA-LIO-SAM
KITTI Odometry Set	1	3.65	3.22	**2.67**	2.89	5.78	5.74
	2	9.53	8.74	10.90	**8.53**	10.25	9.49
	3	4.91	**4.31**	9.25	9.20	21.34	21.17
	4	1.63	1.58	1.85	2.22	1.55	**1.43**
	5	22.94	20.03	17.79	**14.18**	20.08	18.59
	6	17.85	17.92	**8.67**	9.35	12.05	12.29
	7	171.39	165.12	19.76	**13.81**	fail	fail
	8	294.73	230.55	31.30	**24.13**	41.76	34.35
	9	373.20	361.16	145.49	**129.41**	167.86	163.62
KITTI Raw Data Set	1	5.04	4.91	1.70	**0.36**	1.22	1.22
	2	1.76	1.68	1.53	**1.50**	1.51	1.50
	3	13.4	13.24	5.86	**5.83**	7.05	6.98
	4	2.2	2.05	1.27	**1.15**	1.71	1.71

Note: The numbers highlighted in bold indicate the algorithm that achieved the highest performance on each dataset sequence.

**Table 6:** Evaluation of RMSE for LiDAR Odometry with the proposed visual aid module (with loop detection). The visual aid module was incorporated into the three LiDAR odometers that exhibited the most favorable performance in the Kitti test set.

Dataset	Seq No.	LEGO-LOAM [10]	VA-LEGO-LOAM	ISC-LOAM [13]	VA-ISC-LOAM	LIO-SAM [12]	VA-LIO-SAM
KITTI Odometry Set	1	3.74	3.74	**2.67**	2.89	5.77	5.74
	2	7.61	**7.54**	10.89	8.39	8.40	7.97
	3	4.92	**4.31**	9.25	9.20	19.02	19.00
	4	1.76	1.66	1.85	2.22	1.50	**1.42**
	5	22.93	19.87	17.79	**14.18**	20.15	18.68
	6	18.53	18.69	**8.67**	9.35	11.35	12.17
	7	158.65	152.04	19.76	**13.05**	fail	fail
	8	275.88	214.67	31.30	**23.82**	39.79	33.15
	9	376.63	362.46	145.49	**129.04**	166.19	164.48
KITTI Raw Data Set	1	5.04	4.91	1.70	**0.36**	1.22	1.22
	2	1.76	1.68	1.53	**1.50**	1.51	1.50
	3	13.4	13.24	5.86	**5.83**	7.05	6.98
	4	2.2	2.05	1.27	**1.15**	1.71	1.71

Note: The numbers highlighted in bold indicate the algorithm that achieved the highest performance on each dataset sequence.

**Table 7:** The efficacy of the proposed visual aid module evaluated by comparing it with the traditional LiDAR Odometry method to assess the percentage improvement in accuracy without loop detection.

Dataset	Seq No.	Algorithm	Accuracy Improvement (%)	Algorithm	Accuracy Improvement (%)	Algorithm	Accuracy Improvement (%)
Dataset KITTI Odometry Set	1	VA-LEGO-LOAM	11.79	VA-ISC-LOAM	−8.24	VA-LIO-SAM	0.65
	2		8.24		21.73		7.41
	3		12.22		0.63		0.78
	4		2.91		−19.73		7.75
	5		12.68		20.28		7.46
	6		−0.41		−7.86		−1.98
	7		3.66		30.11		-
	8		21.78		22.90		17.75
	9		3.23		11.05		2.52
KITTI Raw Data Set	1	VA-LEGO-LOAM	2.58	VA-ISC-LOAM	78.82	VA-LIO-SAM	0.00
	2		4.55		1.96		0.66
	3		1.19		0.51		0.99
	4		6.82		9.45		0.00
Avg			7.02		12.43		3.67

**Table 8:** The efficacy of the proposed visual aid module evaluated by comparing it with the traditional LiDAR Odometry method to assess the percentage improvement in accuracy with loop detection.

Dataset	Seq No.	Algorithm	Accuracy Improvement (%)	Algorithm	Accuracy Improvement (%)	Algorithm	Accuracy Improvement (%)
Dataset KITTI Odometry Set	1	VA-LEGO-LOAM	0.09	VA-ISC-LOAM	−8.24	VA-LIO-SAM	0.55
	2		0.88		23.01		5.11
	3		12.38		0.63		0.08
	4		5.60		−19.73		5.42
	5		13.33		20.28		7.31
	6		−0.84		−7.86		−7.17
	7		4.17		33.98		-
	8		22.19		23.90		16.69
	9		3.76		11.31		1.03
KITTI Raw Data Set	1	VA-LEGO-LOAM	2.58	VA-ISC-LOAM	78.82	VA-LIO-SAM	0.00
	2		4.55		1.96		0.66
	3		1.19		0.51		0.99
	4		6.82		9.45		0.00
Avg			5.90		5.95		2.42

**Table 9:** This demonstrates the performance of Visual Assist LiDAR Odometry under sensor faults and environmental changes.

Dataset	Seq No.	Environment	VA-LEGO-LOAM	VA-ISC-LOAM	VA-LIO-SAM
KITTI Raw Data set	1	Only LiDAR (Traditional)	5.042	1.698	**1.221**
		Daytime/Urban	4.913	**0.362**	1.215
		Night-time/Urban	4.922	**0.365**	1.218
		Image Sensor Fault	5.042	1.698	**1.215**
	2	Only LiDAR (Traditional)	1.76	1.529	**1.507**
		Daytime/Urban	1.684	**1.496**	1.503
		Night-time/Urban	1.686	**1.497**	1.504
		Image Sensor Fault	1.759	1.529	**1.509**
	3	Only LiDAR (Traditional)	13.375	**5.859**	7.047
		Daytime/Urban	13.241	**5.832**	6.98
		Night-time/Urban	13.297	**5.868**	7.379
		Image Sensor Fault	13.375	**5.859**	6.99
	4	Only LiDAR (Traditional)	2.203	**1.266**	1.71
		Daytime/Urban	2.053	**1.149**	1.705
		Night-time/Urban	2.052	**1.159**	1.706
		Image Sensor Fault	2.203	**1.267**	1.707

Note: The numbers highlighted in bold indicate the algorithm that achieved the highest performance on each dataset sequence.

## Data Availability

Restrictions apply to the availability of these data. Data were obtained from KITTI and are available https://www.cvlibs.net/datasets/kitti/ (accessed on 3 March 2023) with the permission of KITTI.
